# Evaluating the Impact of Family History and Polygenic Risk Scores on Cardiometabolic Disease Risk

**DOI:** 10.21203/rs.3.rs-7142452/v1

**Published:** 2025-08-01

**Authors:** Ebuka Onyenobi, Knightess Oyibo, Michael Zhong, Sally N. Adebamowo

**Affiliations:** University of Maryland School of Medicine; University of Maryland School of Medicine; University of Maryland School of Medicine; University of Maryland School of Medicine

## Abstract

**Background:**

Cardiometabolic diseases (CMD) are a leading cause of morbidity and mortality. While both family history (FH) and polygenic risk scores (PRS) are predictive of CMD risk, few studies have systematically evaluated their independent and joint effects. This study aimed to quantify the individual contributions of FH and PRS, as well as their combined impact on CMD risk.

**Methods:**

We conducted a cross-sectional analysis of 105,633 adults from the All of Us Research Program with available genotypic and FH data. CMDs including type 2 diabetes (T2D), obesity, hypertension (HTN), and coronary artery disease (CAD) were ascertained from electronic health records. FH was derived from self-reported survey responses, and family history scores (FHS) were constructed by weighting the number and degree of affected relatives. PRSs were computed using validated multi-ancestry PRS weights from the PGS catalog. Logistic regression was used to assess associations of FH, FHS and PRS independently and jointly with CMD. We also tested for FHS × PRS interactions and conducted mediation analysis to quantify the proportion of the FHS effect mediated by PRS.

**Results:**

Positive FH was significantly associated with increased risk of all CMDs, with the strongest effect observed for obesity (OR: 2.09, 95% CI: 2.01–2.16). FHS showed the strongest association with T2D (OR: 1.40, 95% CI: 1.38–1.42). Higher PRS values were also associated with elevated disease risk, most strongly for T2D (OR: 2.25, 95% CI: 2.18–2.33). A statistically significant interaction between FHS and PRS was observed for obesity (p = < 0.001). A composite variable combining FH and PRS revealed a stepwise increase in disease odds across risk categories. Mediation analysis indicated that PRS accounted for between 13–17% of the total effect of FHS across all traits.

**Conclusions:**

Both FH and PRS are associated with CMD risk and provide complementary but distinct insights into disease risk. PRS adds predictive value beyond FH and partially mediates its effect. Integration of both measures may enhance risk stratification and guide precision prevention strategies.

## INTRODUCTION

Cardiometabolic diseases (CMDs), a cluster of interconnected conditions including Type 2 diabetes (T2D), obesity, hypertension (HTN), and coronary artery disease (CAD) are among the leading causes of morbidity and mortality worldwide^1^. Studies have shown that having any one of these conditions independently increases the risk of mortality^2,3^ and the presence of multiple CMDs further amplifies this risk in an additive manner^4^. The prevalence of CMDs is rising rapidly^5^. In the United States, the estimated prevalences in 2023 were 10% for T2D, 35% for obesity, 27% for HTN, and 4.5% for CAD^6^. CMDs arise from a dynamic interplay between environmental and lifestyle factors and an individual’s genetic predisposition. Among modifiable lifestyle risk factors, elevated Body Mass Index (BMI), and insufficient physical activity have been consistently implicated in the development and progression of these conditions^7,8^. Genetic risk factors also play a crucial role in the development of CMDs, influencing individual susceptibility through inherited variations in multiple genes^9,10^.

Prior to the advent of high-throughput genotyping technologies, family history (FH) served as a clinical proxy for genetic risk^11^. FH remains a strong predictor of CMDs, capturing the combined influence of genetic, behavioral, and environmental factors. Multiple studies have demonstrated associations between FH and T2D, obesity, HTN, and CAD^12–15^. However, its utility is limited by factors such as family size, disease prevalence, and susceptibility to recall bias^16,17^. Advances in genomics have enabled the development of polygenic risk scores (PRSs) which aggregate the effects of common genetic variants to quantify an individual’s risk of disease. PRSs are derived from genome-wide association studies (GWAS) and have been applied to predict risk across a range of conditions, including CMDs^18–21^. As a scalable and objective measure, PRSs have the potential to enhance risk stratification beyond what FH alone can offer.

In this study, we evaluated the associations of FH and PRS with a range of common CMDs, including T2D, obesity, HTN, and CAD, and investigated the extent to which these familial and genetic indicators of risk contribute independently or interactively to disease susceptibility in a large, ancestrally diverse cohort.

## METHODS

### Study Population

We conducted a cross-sectional study using data from unrelated individuals in the All of Us dataset^22^. The study included participants with available FH and genotypic data resulting in an initial sample of 105,633 individuals. After restricting the cohort to adults aged 18 years and older, the final analytic sample comprised 103,566 participants. The global continental ancestry composition of the cohort was as follows: 74.13% European, 10.99% Admixed American, 10.49% African, 2.51% East Asian, 1.46% South Asian, and 0.39% Middle Eastern.

### Phenotype Definition

Phenotypic definitions were based on linked electronic health record (EHR) data, which were used to ascertain case and control status for each CMD trait of interest, including T2D, obesity, HTN, and CAD. T2D, obesity, and CAD cases were defined as ≥ 1 diagnostic code in the EHR, and HTN was defined as ≥ 1 diagnostic code in the EHR and documentation of antihypertensive medication use, consistent with Electronic Medical Records and Genomics (eMERGE) network recommendations^23,24^. Controls were defined as individuals with no recorded diagnosis of the corresponding CMD phenotype at the time of data extraction.

### Family History Assessment

We obtained FH information from responses to the Family Health History survey in the All of Us data. Participants were asked: “Who in your family has had [condition]? Select all that apply.” Conditions of interest included T2D, obesity, HTN, and CAD. A positive family history was defined as the participant reporting at least one affected relative (including mother, father, son, daughter, sibling, or grandparent). For sensitivity analysis, we categorized relatives into first-degree relatives (mother, father, son, daughter, sibling) and second-degree relatives (grandparents). To quantify FH burden, we also constructed a continuous family history score (FHS), which incorporated both the number and degree of relatedness of affected relatives. Each first-degree relative reported as affected was assigned a weight of 0.5, while each second-degree relative was assigned a weight of 0.25 reflecting approximate genetic relatedness^25^. The FHS was calculated as the weighted sum of affected relatives for each condition, resulting in a continuous measure of familial burden consistent with approaches used in prior studies^26^. The FHS was standardized (z-scored) within each condition to enable comparability across analyses.

### Polygenic Risk Score

Genotype data from the All of Us Research Program Controlled Tier V7 whole-genome sequencing (WGS) was used for this analysis. PRSs for the relevant CMD traits were obtained from the PGS catalog. We selected previously published multi-ancestry PRS that have been validated in diverse populations including: T2D (PGS002308)^21^, BMI (PGS004994)^27^, systolic blood pressure (SBP) (PGS003968)^28^, and CAD (PGS005092)^29^. These scores were chosen to ensure generalizability across the diverse ancestry groups represented in our cohort. SBP-PRS (PGS003968) was used as a proxy for HTN due to prior evidence demonstrating that SBP polygenic scores exhibit stronger predictive utility for HTN status compared to binary HTN PRSs^28^. Individual PRSs were computed in the target dataset using the --score function in PLINK 2, applying the published SNP weights.

### Association Analysis

We assessed the independent and joint associations of FH and PRS with each CMD using multivariable logistic regression. For each condition, we fit three sets of models: one including binary FH as the primary predictor, one including FHS as the primary predictor, and one including PRS as the primary predictor, and a combined model including both FHS and PRS. We also tested statistical interactions between PRS and FHS by including an interaction term (PRS × FH) in the regression models. All models were adjusted for age, sex, BMI, ancestry and the top 10 principal components (PCs), except for the obesity model, in which BMI was excluded.

Additionally, PRS was analyzed as a categorical variable, grouped into three risk strata: low (≤ 20th percentile), intermediate (20th–80th percentile), and high (≥ 80th percentile). Associations between PRS categorical variable and each CMD was evaluated with the intermediate category as the reference group. To assess the combined influence of PRS and FH, we created a six-level composite variable reflecting both PRS category and FH status. This variable included the following groups: PRS_low_FH_no_, PRS_low_FH_yes_,PRS_intermediate_FH_no_, PRS_intermediate_FH_yes_, PRS_high_FH_no_, PRS_high_FH_yes_. This combined variable was entered into a logistic regression model to evaluate the joint effects of PRS and FH on CMD outcomes. By using the PRS_intermediate_FH_no_ group as the reference category, we were able to quantify the incremental risk associated with higher or lower genetic risk and the presence or absence of a FH. We evaluated the discriminative ability of PRS and FH using receiver operating characteristic (ROC) curve analysis. The area under the curve (AUC) was used to quantify model performance

### Mediation Analysis

We conducted mediation analysis to evaluate whether PRSs mediated the association between FHS and CMD outcomes. Models were adjusted for age, sex, BMI, ancestry and the top 10 PCs, except for the obesity model, in which BMI was excluded. PRS and FHS were modeled as continuous variables. We estimated the mediated effects, direct effects, and total effects using 1,000 Monte Carlo simulations to generate quasi-Bayesian 95% confidence intervals. All mediation analyses were performed using the mediation package in R^30^. All analyses were performed using R version 4.0.3.

## RESULTS

### Study Characteristics

In our study, the prevalence of CMDs among participants was 15.37% for T2D, 22.4% for obesity, 30.83% for HTN, and 9.59% for CAD. Across all CMD outcomes, the cases were generally older than controls (Table 1). T2D, HTN and CAD had a larger proportion of males compared to controls, while obesity cases had a larger proportion of females. BMI values were consistently higher among cases across all CMD outcomes. T2D cases had a mean (standard deviation [SD]) BMI of 33.77 (SD = 8.22), while controls had a mean of 28.64 (6.81). HTN and CAD cases had mean BMIs of 31.68 (7.59), and 30.36 (6.63), respectively, compared to 28.19 (6.75), and 29.22 (7.22) among their respective controls. A positive FH of CMDs was more prevalent among cases than controls. The prevalence of FH was higher in cases compared to controls: 41% vs 27% for T2D, 28% vs 15% for obesity, 60% vs 54% for HTN, and 33% vs 21% for CAD (Table 1).

### Association of Family History and Polygenic Risk Scores with Cardiometabolic Traits

**Figure 1:**
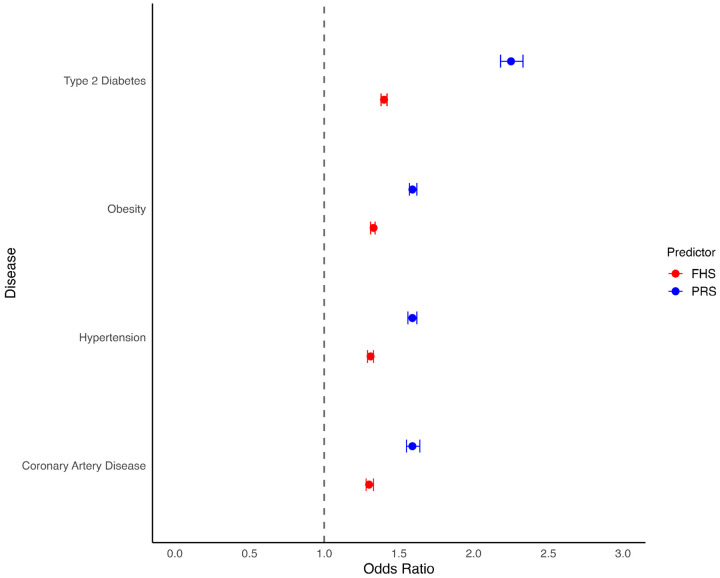
Association between Polygenic Risk Score (PRS), Family History Score (FHS) and Cardiometabolic diseases. We evaluated the independent association of PRS and FHS with type 2 Diabetes, obesity, hypertension and coronary artery disease. Forest plot showing Odds Ratios and 95% confidence intervals.

The strongest association between positive FH and CMD was observed for obesity, with individuals having a positive FH showing more than twice the odds of developing the condition (OR 2.09, 95% CI: 2.01–2.16). FH was also significantly associated with T2D (OR 1.97, 95% CI: 1.89–2.05), CAD (OR 1.85, 95% CI: 1.76–1.94) and HTN (OR 1.44, 95% CI: 1.39–1.48). Associations were stronger among individuals reporting a first-degree FH, with the highest odds seen for T2D (OR 2.24; 95% CI: 2.15–2.33), followed by obesity (OR 2.14; 95% CI: 2.07–2.22), CAD (OR 2.06; 95% CI: 1.96–2.16), and HTN (OR 1.51; 95% CI: 1.46–1.56) (Figure S1). Similarly, the FHS was associated with increased odds per standard deviation for T2D (1.40, 95% CI: 1.38–1.42), obesity (OR 1.33, 95% CI: 1.31–1.34), HTN (OR 1.31, 95% CI: 1.29–1.33) and CAD (OR 1.30, 1.28–1.33) (Fig. 1). Adjusting FHS effect size for PRS resulted in modest attenuation of effect estimates, ranging from 3–4% (Table S1).

PRSs were also significantly associated with elevated risk of disease across all conditions. Higher PRS values were linked to increased odds per standard deviation for T2D (OR 2.25, 95% CI: 2.18–2.33), obesity (OR 1.59, 95% CI: 1.57–1.62), HTN (OR 1.59, 95% CI: 1.56–1.62), and CAD (OR 1.59, 95% CI: 1.55–1.64) (Fig. 1). When stratified by PRS categories, individuals in the low PRS group had significantly lower odds of T2D compared to those in the intermediate group (OR 0.43, 95% CI: 0.40–0.46). In contrast, individuals in the high PRS group had higher odds of T2D (OR 2.13, 95% CI: 2.00–2.26). For obesity, the high PRS group had increased odds relative to the intermediate group (OR 1.83, 95% CI: 1.77–1.90). Individuals in the high PRS group also had higher odds of HTN (OR 1.82; 95% CI: 1.74–1.89), and CAD (OR 1.90; 95% CI: 1.81–2.00) (Figure S2). Adjusting PRS models for FHS led to attenuation of associations ranging from 2–7% (Table S1). Interaction between FHS and PRS was statistically significant for obesity (p = < 0.001).

### Joint Associations of Polygenic Risk Scores and Family History

**Figure 2:**
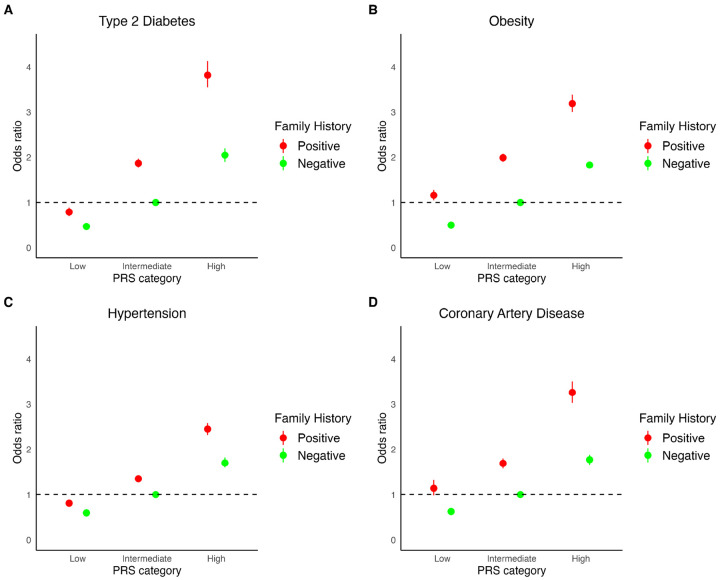
Joint Effect of Polygenic Risk Score (PRS) and Family History on Cardiometabolic Diseases. We evaluated the joint effects of PRS and family history score on type 2 diabetes, obesity, hypertension, and coronary artery disease. Participants were categorized into low, intermediate, and high PRS groups, and odds ratios were estimated within each category, further stratified by family history status.

Individuals with both a positive FH and a high PRS had significantly higher odds of disease compared to those with negative FH and an intermediate PRS for T2D (OR 3.82, 95% CI: 3.55–4.13), obesity (OR 3.19, 95% CI: 3.00–3.39), HTN (OR 2.45, 95% CI: 2.32–2.58), and CAD (OR 3.26, 95% CI: 3.03–3.50). Conversely, individuals with negative FH and a low PRS had reduced odds of disease compared to those with negative FH and an intermediate PRS: T2D (OR 0.47, 95% CI: 0.43–0.50), obesity (OR 0.50, 95% CI: 0.48–0.53), HTN (OR 0.60, 95% CI: 0.56–0.64), and CAD (OR 0.63, 95% CI: 0.58–0.69). Notably, individuals with an intermediate PRS and a positive FH exhibited similar risk compared to those with a high PRS but negative FH (Fig. 2).

### Predictive Utility of Polygenic Risk Scores and Family History

**Figure 3:**
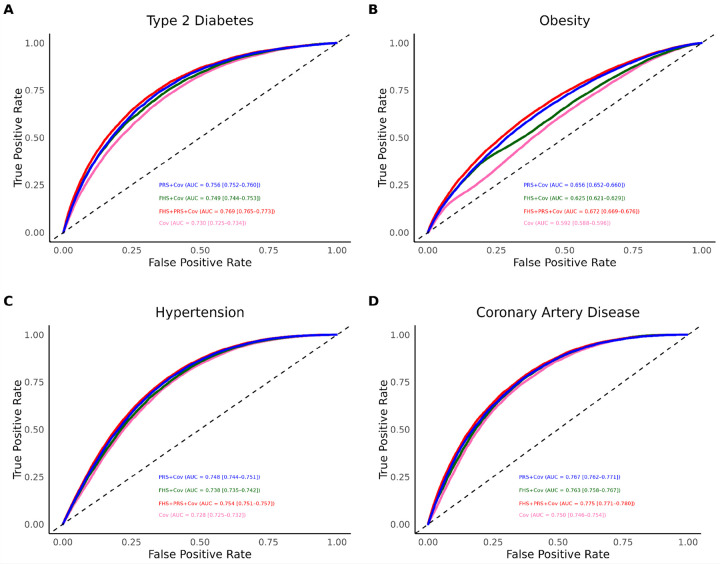
Discriminatory Performance of Polygenic Risk Score (PRS) and Family History Score (FHS) Across Cardiometabolic Diseases. We assessed the predictive performance of PRS and FHS for type 2 diabetes, obesity, hypertension, and coronary artery disease using receiver operating characteristic (ROC) curve analysis. Model discrimination was quantified by the area under the ROC curve (AUC), with comparisons made between models incorporating PRS alone, FHS alone, and both combined.

For T2D, the baseline model including only covariates showed good discrimination (AUC 0.730, 95% CI: 0.25–0.734). The addition of FHS (AUC 0.749, 95% CI: 0.744–0.753), and PRS (AUC 0.756, 95% CI: 0.752–0.760) offered an increase to AUC. Notably, the combination of PRS and FHS resulted in the highest AUC of 0.769 (95% CI: 0.765–0.773). A similar trend was observed for obesity, though overall AUCs were lower. The covariate-only model had an AUC of 0.592 (95% CI: 0.588–0.596) which increased with the addition of FHS 0.625 (95% CI, 0.621–0.629) and PRS 0.656 (95% CI, 0.652–0.660). The combined model of PRS and FHS achieved the highest AUC of 0.672 (95% CI, 0.669–0.676). For HTN, the baseline AUC was 0.728 (95% CI, 0.725–0.732). This increased to 0.738 (95% CI: 0.735–0.742) for FHS and 0.748 (95% CI: 0.744–0.751) for PRS. The combination of PRS and FHS provided the highest discrimination with an AUC of 0.754 (95% CI: 0.751–0.757). CAD had the highest baseline AUC at 0.750 (95% CI, 0.746–0.754). Adding FHS and PRS raised the AUC to 0.763 (95% CI: 0.758–0.775) and 0.767 (95% CI: 0.762–0.771) respectively, with the highest value observed in the combined model (AUC 0.775, 95% CI: 0.771–0.780) (Fig. 3).

### Polygenic Risk Scores as a Mediator of the Association between Family History and Cardiometabolic conditions

The total effect (TE), and mediated effect (ME) were estimated for each trait to quantify both the direct and indirect components of familial risk using the FHS. For T2D, the TE of FHS was 0.039 (95% CI: 0.037–0.04) and an ME of 0.006 (95% CI: 0.006–0.007), indicating that 16% of the total effect of FH on T2D risk was mediated through the PRS. Similarly, for obesity, the TE and ME were 0.052 (95% CI: 0.049–0.05) and 0.009 (95% CI: 0.08 − 0.009), respectively, corresponding to 17% of the TE being mediated. In the case of HTN, the TE and ME were 0.051 (95% CI: 0.048–0.05) and 0.009 (95% CI: 0.008–0.01), suggesting that 17% of the FH effect on HTN was mediated by PRS. Lastly, for CAD, the TE and ME were 0.023 (95% CI: 0.021–0.024) and 0.003 (95% CI: 0.0027–0.0033) representing 13% of the TE mediated by PRS (Table 2).

## DISCUSSION

FH and PRS are both valuable tools for disease risk prediction, yet few studies have evaluated their independent and joint contributions to CMD. It remains unclear to what extent FH and PRS capture overlapping or distinct components of risk and whether their effects on CMD susceptibility are modified by one another. In this study, we examined the associations of FH and PRS with T2D, obesity, HTN, and CAD while also exploring potential interaction and mediation effects. We found that both FH and PRS were independently associated with increased odds of each CMD. Moreover, individuals with both positive FH and a high PRS were at substantially elevated risk, underscoring the additive contribution of familial and genetic risk to CMD susceptibility.

Consistent with previous literature^14,31–33^, a FH of CMDs was significantly associated with increased disease risk across all phenotypes with the strongest association observed for obesity even after adjusting for PRS. Prior studies have shown that a positive family history can increase the risk of CAD by approximately 50%^34^, reinforcing FH as a robust risk indicator that captures a wide array of environmental, molecular, and social determinants of disease risk^35^. PRS were also significantly associated with CMD traits; per SD increase in PRS, the odds of T2D and obesity increased by 2.25 and 1.59 times respectively, consistent with previous studies^36,37^. Stratified analyses revealed a gradient of risk across PRS categories, indicating the utility of PRS in stratifying individuals based on genetic liability. Notably, we observed evidence of interaction between FH and PRS for obesity, suggesting that FH may modify the association between PRS and this outcome. This may reflect underlying gene - environment correlations within families, where shared behaviors such as dietary patterns, physical activity levels and socioeconomic conditions interact with genetic predisposition to influence disease expression^38^.

Individuals with both a positive family history and high PRS had significantly greater odds of CMD than those with neither risk factor. The combined effect was nearly threefold for obesity and T2D, and nearly double for CAD and HTN. This is consistent with a previous study demonstrating that incorporating PRS with FH improves disease risk prediction for T2D, HTN and depression^39^. Similarly, studies in various cancers have identified the utility of integrating FH and PRS for improved risk stratification^40–42^. Interestingly, individuals with negative FH but a high PRS had a comparable risk to those with FH and low PRS, suggesting that either risk factor independently confers substantial susceptibility and reflects different aspects of disease risk. This observation is aligned with findings that further support the complementary roles of FH and PRS in risk prediction^43^. In terms of predictive utility, we found that while baseline models incorporating traditional covariates demonstrated reasonable discrimination, the addition of either FHS or PRS improved risk prediction. Importantly, the combination of both FHS and PRS consistently yielded the highest AUC values across all four CMDs, suggesting that integrating these two sources of information enhances the accuracy of risk stratification as reported in previous studies^44^. These findings underscore the clinical importance of incorporating both FH and genetic risk into comprehensive risk assessment frameworks^45^.

Mediation analysis further explored the pathway through which FH may influence CMD risk. Across all traits, we found that PRS mediated a proportion of the total effect of FHS. For example, in our study, PRS accounted for between 12–17% of the total effect of a FHS. This is comparable to previous studies where partial mediation of FH effects by PRS has been reported in other complex diseases such as schizophrenia with 17.4% mediation^46^ and chronic obstructive pulmonary disease with 16.5% mediation^44^. These results suggest that while PRS captures some of the genetic component embedded within FH, much of the FH effect remains unexplained by current PRS models. These residual effects may be attributable to rare variants, gene environment interactions, epigenetic inheritance, or unmeasured familial exposures and behaviors^47,48^. Importantly, these findings highlight the limitations of PRS in fully capturing inherited risk^49,50^. Conversely, FH continues to serve as a valuable, holistic measure of genetic and environmental risk, offering clinical utility independent of PRS. While PRS are valuable tools for stratifying genetic risk and hold promise for personalized prevention strategies, they should be viewed as complementary rather than replacements for FH assessments in clinical settings. Integrating both measures can enhance risk prediction and inform tailored intervention strategies.

This study leveraged a large sample of diverse ancestry individuals from the All of Us cohort, enhancing statistical power. A key strength lies in the use of publicly available multi-ancestry PRS, which are known to improve predictive performance in diverse populations. PRS were derived from consistent methodologies allowing for a robust comparison of genetic risk across traits. Additionally, CMD diagnoses were derived from structured electronic health records rather than self-reported data, reducing misclassification bias and improving phenotypic accuracy. However, several limitations should be noted. FH was self-reported, introducing potential for measurement error and recall bias. In addition, FH data collection was limited to a subset of relatives, excluding second-degree relatives such as uncles, aunts, nephews, nieces, and grandchildren, likely leading to an underestimation of individuals with a positive family history.

## CONCLUSIONS

FH remains a good indicator that captures both inherited genetic risk and shared environmental exposures, while PRS offers a complementary, quantifiable measure of genetic liability. Rather than serving as a replacement, PRS should be viewed as augmenting the information provided by FH. Integrating both metrics may enhance risk prediction models and improve individualized risk communication and clinical decision-making. Future research should focus on the development and validation of integrated risk models that combine genetic, familial, and environmental factors to more comprehensively capture the determinants of disease susceptibility and promote more equitable and precise approaches to prevention and care.

## Supplementary Material

Supplementary Files

This is a list of supplementary files associated with this preprint. Click to download.

- suplementaryfamilyhistory.docx

## Figures and Tables

**Table 1:** Participant Characteristics by Cardiometabolic Disease Status

	Type 2 diabetes N = 103,540		Obesity N = 103,379		Hypertension N = 85,889		Coronary artery disease N = 103,563	
	Cases	Controls	Cases	Controls	Cases	Control	Cases	Controls
**N**	13,792	89,748	23,167	80,212	26,478	59,411	9,927	93,636
**Age (mean (SD))**	55.83 (12.74)	52.43 (17.03)	50.50 (14.24)	53.13 (17.25)	56.37 (12.36)	48.42 (16.90)	63.52 (10.64)	51.99 (16.64)
**Male (n (%))**	5,785 (41.94)	30,511 (34)	7,189 (31.10)	29,088 (36.26)	11,595 (43.79)	18,282 (30.77)	5,737 (57.79)	30,562 (32.64)
**BMI (Kg/m** ^**2**^ **) (mean (SD))**	33.77 (8.22)	28.64 (6.81)	36.07 (7.15)	27.36 (5.94)	31.68 (7.59)	28.06 (6.70)	30.36 (6.63)	29.22 (7.28)
**Positive FH (n (%))**	5,684 (41.21)	23,838 (26.56)	6,559 (28.31)	12,356 (15.40)	15,820 (59.75)	32,223 (54.24)	3,318 (33.42)	19,620 (20.95)

BMI, body mass index; FH, family history

**Table 2:** Mediation of Family History Effects on Cardiometabolic Diseases Through Polygenic Risk Scores

	Total Effect (95%CI)	Direct Effect (95% CI)	Mediated Effect (95% CI)	Proportion mediated (95% CI)
Type 2 Diabetes	0.039 (0.037–0.04)	0.033 (0.031–0.035)	0.006 (0.006–0.007)	0.16 (0.15–0.18)
Obesity	0.052 (0.049–0.05)	0.043 (0.041–0.05)	0.009 (0.008–0.009)	0.17 (0.15–0.18)
Hypertension	0.051 (0.048–0.05)	0.042 (0.039–0.046)	0.009 (0.008–0.01)	0.17 (0.16–0.19)
Coronary Artery Disease	0.023 (0.021–0.024)	0.020 (0.018–0.021)	0.003 (0.0027–0.0033)	0.13 (0.12–0.15)
